# Development of a loop-mediated isothermal amplification (LAMP) assay for the detection of *Cyclospora cayetanensis*

**DOI:** 10.1016/j.fawpar.2026.e00337

**Published:** 2026-05-05

**Authors:** Mahdid Meymandy, Nadine Adam, Nathalie Corneau, Brent R. Dixon

**Affiliations:** Bureau of Microbial Hazards, Food and Nutrition Directorate, Health Canada, 251 Sir Frederick Banting Driveway, Ottawa, Ontario K1A 0K9, Canada

**Keywords:** *Cyclospora cayetanensis*, Parasite, Detection, LAMP, Food, Clinical

## Abstract

Many surveillance studies worldwide have reported the presence of *Cyclospora cayetanensis* oocysts, or DNA, on a variety of fruits and vegetables using either microscopy or PCR-based methods respectively. However, the true prevalence of *C. cayetanensis* on foods is still largely unknown due to a lack of efficient, accessible, and standardized detection methods. The present study describes, for the first time, a LAMP assay for the detection of *C. cayetanensis* DNA, which was found to be rapid, sensitive and specific. Using DNA extracted from a *C. cayetanensis*-positive stool specimen, LAMP products stained with SYBR Green I fluoresced down to at least a 1:10^7^ dilution. Colorimetric, pH-based visualization of the LAMP reaction with phenol red was found to be considerably less sensitive than with SYBR Green I. While further work is required, the LAMP assay described here may be useful in surveillance studies and outbreak investigations involving food and environmental samples, as well as in screening clinical specimens.

## Highlights

• A novel LAMP assay was developed for the detection of *Cyclospora cayetanensis* DNA.
• This *Cyclospora* LAMP assay is rapid, cost-effective, sensitive and specific.
• UV visualization of LAMP products using SYBR Green I was the most sensitive method.
• This method has potential applications in food, environmental and clinical testing.

## 1 Introduction

*Cyclospora cayetanensis* is a relatively common enteric parasite in humans worldwide (Almeria et al., 2019; Dixon et al., 2016). Cyclosporiasis can result in profuse and prolonged diarrhea and a variety of other symptoms including abdominal pain, nausea, vomiting, fatigue, fever and loss of appetite (Ortega and Sanchez, 2010). Although *C. cayetanensis* is thought to be primarily a waterborne parasite; foodborne outbreaks have been reported annually in North America since the mid-1990s; with fresh produce generally being implicated (Almeria et al., 2019; Dixon et al., 2016).

Although numerous surveillance studies have reported the presence of *C. cayetanensis* oocysts, or DNA, on a variety of fruits and vegetables (Almeria et al., 2023; Dixon, 2016), knowledge of the true prevalence of *C. cayetanensis* on foods has been hampered by a lack of efficient, accessible, and standardized detection methods. As with other foodborne parasitic infections, some of the same methods used in the clinical diagnosis of cyclosporiasis have been evaluated and adapted for foods.

Most of the published methods for the detection of *C. cayetanensis* on fresh produce involve agitation of the fruits or vegetables in water or buffer to elute the oocysts, often in combination with detergents (Dixon et al., 2016); followed by concentration using centrifugation and/or flotation. Testing has generally involved the use of microscopy for the detection of oocysts; or PCR-based methods for the detection of *C. cayetanensis* DNA; both of which have important limitations. Microscopy; for example; is very time-consuming; and requires a high level of expertise. Unless the oocysts can be purified; their detection and identification is often very challenging due to extraneous debris on the microscope slide. Various stains; including modified acid-fast stain; have been used to mitigate this issue; and the autofluorescence of *C. cayetanensis* oocysts under UV light has also been utilized. However; other coccidian parasites also stain with acid-fast stain and can autofluoresce; making identification difficult (Ortega and Sanchez, 2010). Furthermore; there are currently no commercially available monoclonal antibodies specifically targeting the *C. cayetanensis* oocyst wall; and DNA aptamers for the detection of *C. cayetanensis* have only recently been developed (Bruno et al., 2023). PCR technology has also been used extensively for the detection of *C. cayetanensis* DNA in food and environmental samples (Durigan et al., 2024), but requires specialized equipment, and the entire procedure including DNA extraction, amplification by thermocycling, and detection of amplicons by electrophoresis (in the case of conventional PCR), is relatively time-consuming. Although some of these limitations can be addressed using various modifications, such as real-time PCR, PCR-based tests are expensive to run and are not always widely accessible in low-income countries.

Loop-mediated isothermal amplification (LAMP), originally developed by Notomi et al. (2000); is a novel and rapid method for the isothermal amplification of target DNA with high sensitivity and specificity (Soroka et al., 2021; Yang et al., 2024). LAMP amplification can often be completed within one hour, and has a sensitivity similar to that of nested PCR (Notomi et al., 2015). This technology is also relatively inexpensive as specialized equipment needed for thermal cycling and electrophoresis are not required. LAMP may; therefore; be more practical for use in point-of-care testing of parasite-caused neglected tropical diseases (García-Bernalt Diego et al., 2021). It is also well suited for use with microfluidic lab-on-a-chip technologies for detection of pathogens including parasites and viruses (Chen et al., 2023; Malic et al., 2022; Yang et al., 2024; Zhang et al., 2019).

Whereas PCR amplification typically involves only two primers, LAMP uses four to six primers, including forward and backward internal primers, forward and backward external primers, and the optional forward and backward loop primers which make it considerably more sensitive and specific (Soroka et al., 2021). In the presence of Bst DNA polymerase; which has a high strand displacement activity at 60–65 °C; the internal and external primers create a dumbbell-like structure which then serves as a template for further amplification (Soroka et al., 2021; Yang et al., 2024). Loop primers, complementary to this structure, can be added to increase the number of “starting points” up to a total of eight amplified DNA sequences (Soroka et al., 2021). LAMP can generate up to 10^9^ copies of the amplified DNA in less than one hour (Soroka et al., 2021; Yang et al., 2024). Since the original development of the LAMP assay; numerous other forms of LAMP amplification have been developed; including reverse-transcriptase LAMP; real time LAMP; and multiplex LAMP (Soroka et al., 2021; Wong et al., 2018).

LAMP products can be monitored by a variety of methods, including turbidimetry, fluorometry and colorimetry (Yang et al., 2024). In the latter two methods; results can be immediately visualized with the naked eye; eliminating the need for time-consuming electrophoretic separation as in PCR (Soroka et al., 2021). Electrophoresis can; however; be used to visualize LAMP amplification products; but since these products appear as ladder-like patterns on agarose gels; the identification of a target based on band size; as in PCR; is not possible (Wong et al., 2018).

Although numerous LAMP assays have been published for the detection of important food and waterborne protozoan parasites, including: *Giardia duodenalis*, *Cryptosporidium* spp. and *Toxoplasma gondii* in clinical and environmental samples (Karanis et al., 2007; Lalonde et al., 2021; Sheng et al., 2023), a LAMP assay for the detection of *C. cayetanensis* has not previously been described. Given the importance of *C. cayetanensis* as an emerging foodborne pathogen worldwide, and the scarcity of standardized methods for its detection, there is an urgent need for novel and effective testing methods. Here, we report on the development of a LAMP assay for the rapid, sensitive and specific detection of *C. cayetanensis* DNA, with possible applications in food, environmental and clinical testing.

## 2 Materials and methods

### 2.1 Human stool specimens

A frozen human stool specimen, containing *Cyclospora cayetanensis* oocysts, was generously provided by Dr. Rebecca Guy, National Microbiology Laboratory, Public Health Agency of Canada. A real time PCR assay targeting the 18S gene (Murphy et al., 2017) was used to determine and quantitate *Cyclospora* positivity in this sample (13,000 gene copies/μL). Similarly, a frozen human stool specimen demonstrated to be negative for *C. cayetanensis* using the same method was used as a control.

### 2.2 Extraction of DNA from stool specimens

DNA was extracted from both the *Cyclospora*-positive and negative human stool specimens using a QIAamp Fast DNA Stool Mini Kit (Qiagen, Inc., Toronto, ON) as per the manufacturer's instructions, with an additional step of five freeze-thaw cycles of 1 min in liquid nitrogen and 1 min in boiling water prior to adding proteinase K. Serial dilutions of the DNA were prepared in double distilled water as follows: extracted DNA was quantified using a BioDrop spectrophotometer (MBI, Dorval, QC) and then serially diluted, starting from an initial DNA concentration of 6.57 ng/μL (undiluted) in the positive stool and 1.07 ng/μL (undiluted) in the negative stool, down to a 1:10^9^ dilution.

### 2.3 Primer design

Multicopy organellar DNA such as mitochondrial genomes have been informative for detection and genetic traceback analysis in other parasites (Cinar et al., 2015, Cinar et al., 2020). The *C. cayetanensis* mitochondrial genome is 6274 bp in length; with a 33% GC content (Cinar et al., 2015) and a high degree of conservation (Barratt et al., 2023). Molecular Evolutionary Genetics Analysis X (MEGA X) software (2026) was used to align 35 different *C. cayetanensis* mitochondrial genome sequences downloaded from the NCBI database (Supplementary Table 1); and highly conserved regions were identified. Using the NEB LAMP Primer Design Tool (2026); five candidate sets of primers were initially generated; but only one set had two loop primers. While loop primers are not necessary for a LAMP reaction; the addition of loop primers improves the efficiency and sensitivity of the reaction; and increases the speed (Soroka et al., 2021). The designed LAMP primer set for detection of *C. cayetanensis* covers a 225 bp portion of the mitochondrial genome, spanning the 3407–3631 bp region (GenBank accession no. KP231180.1).

Primers consisted of forward (F3) and backward (B3) outer primers, forward (FIP) and backward (BIP) inner primers, a loop forward primer (LF), and a loop backward primer (LB). The LAMP primer sequences used are shown in Table 1.

**Table 1:** Loop-mediated isothermal amplification (LAMP) primer sequences, spanning the 3407–3631 bp region of the mitochondrial genome for the detection of *Cyclospora cayetanensis.*

Primer name (abbreviation)	DNA Sequence (5′ to 3′)
Forward Outer Primer (F3)	GGATATAATTTGGTAGTGGAACAT
Backward Outer Primer (B3)	GCTAAACTTCCCTTATTTTTTGTCT
Forward Inner Primer (FIP)	GATCACGCATGGATTCAGTGTCTGGAAGACGGAATCGTT
Backward Inner Primer (BIP)	CGGTCCCTGGCTGAATTTTATGAAACACACTTCCCTTCTCG
Loop Forward Primer (LF)	CCAGGACTACCTGACGCTTAGT
Loop Backward Primer (LB)	CCCAGGCTGGTTCAAAAAGTCA

### 2.4 Synthetic gene fragment as a positive control

As a positive control, a 501 bp sequence DNA template was designed to mimic the 3300–3800 bp region of the *C. cayetanensis* mitochondrial genome. This synthetic template was purchased as a gBlocks™ Gene Fragment from Integrated DNA Technologies (Coralville, IA), and is hereafter referred to as the Cyc-Mit gBlock (Supplementary Table 2).

### 2.5 Loop-mediated isothermal amplification

The LAMP assay was carried out as per that of an automated sample-to-answer centrifugal microfluidic system for the rapid molecular diagnostics of SARS-CoV-2 (Malic et al., 2022) with the following modifications: A 10× mixture of LAMP primers was prepared in nuclease-free water including F3 and B3 (2 μM each), FIP and BIP (16 μM each), LF and LB (10 μM each). A 2× isothermal amplification buffer was prepared containing 20 mM (NH_4_)_2_SO_4_, 100 mM KCl, 16 mM MgSO_4_, 80 mM GuHCl, and 0.2% Tween 20 (all obtained from Sigma-Aldrich, Oakville, ON). A final 25 μL LAMP reaction included 12.5 μL 2× isothermal amplification buffer (pH 8.5), 2.5 μL 10× primer mix, 1.0 μL WarmStart Bst 2.0 (New England Biolabs, Ipswich, MA), 0.5 μL Antarctic Thermolabile UDG (New England Biolabs), 1.4 mM dNTPs (Qiagen), 280 μM dUTP (New England Biolabs), and template DNA (1 μL of either stool specimen or Cyc-Mit gBlock). One μL of double distilled water was added for the negative control reaction. Isothermal amplification was performed by incubation of the tubes in an Eppendorf Mastercycler Nexus X2 thermocycler (Eppendorf) at 63 °C for 30 min followed by heating at 80 °C for 5 min to terminate the reaction.

### 2.6 Visualization of LAMP products

For fluorescent visualization of LAMP products, 1 μL of a 1000× concentrate of SYBR Green I dye (Invitrogen, Carlsbad, CA) was added to the inside of the tube cap. Following the incubation, tubes were inverted, tapped on the lab bench and vortexed to ensure that the SYBR Green I dye was fully mixed with the LAMP products. Tubes with SYBR Green I were immediately observed under UV light using a UV Transilluminator (Ultra-Violet Products, Inc., San Gabriel, CA), with a bright green colour indicating a positive reaction. For colorimetric visualization, phenol red was added directly to the master mix prior to incubation at a final concentration of 100 μM per 25 μL reaction. Tubes with phenol red were observed with the naked eye, with a change from pink to yellow indicating a positive reaction. All reaction tubes were then photographed using a digital camera.

LAMP products (5 μL) were also electrophoresed at 110 V for 60 min on a 2% agarose gel stained with 5 μL of GelRed nucleic acid stain to visualize the ladder-like patterns in positive samples. GelPilot Mid Range Ladders (Qiagen, Inc., Toronto, ON) were added to the gels to estimate amplicon sizes. Stained gels were visualized and photographed using a Gel Doc EZ Imager (Bio-Rad, Mississauga, ON).

### 2.7 Specificity of LAMP assay against other parasites and foodborne pathogens

To evaluate the specificity of the *C. cayetanensis* LAMP assay, nucleic acids from several bacterial species, including common foodborne pathogens, as well as from hepatitis A and norovirus (Table 2), were extracted from pure laboratory cultures or from fecal specimens obtained from either ATCC or from colleagues within the Bureau of Microbial Hazards, Health Canada. Nucleic acid extraction methods for bacterial pathogens included DNA boil protocols and the Invitrogen PureLink kit (Invitrogen, Carlsbad, CA). RNA was extracted from norovirus fecal filtrate and hepatitis A cell culture supernatant using the Viral RNA Mini Kit (Qiagen, Mississauga, ON, Canada) according to the manufacturer's protocol. Reverse transcription (RT)-PCR was conducted using a One-Step RT-PCR kit (Qiagen) according to the manufacturer's instructions, and primers as described previously for norovirus (Nasheri et al., 2017) and SH-Poly primers as described previously for hepatitis A virus (Guevremont et al., 2006).

**Table 2:** Inclusivity/exclusivity panel of pathogens tested using the *Cyclospora cayetanensis* loop-mediated isothermal amplification (LAMP) assay.

Pathogen tested	Sample type	LAMP results
*Cyclospora cayetanensis*	Isolated DNA	Positive
*Eimeria acervulina*	Isolated DNA	Negative
*Cryptosporidium parvum*	Isolated DNA	Negative
*Giardia duodenalis*	Isolated DNA	Negative
*Toxoplasma gondii*	Isolated DNA	Negative
*Bacillus subtilis* ATCC 6051	Isolated DNA	Negative
*Shewanella putrefaciens*	Isolated DNA	Negative
*Serratia fonticola*	Isolated DNA	Negative
*Vibrio parahaemolyticus* ATCC 17802	Isolated DNA	Negative
*Enterobacter kobei*	Isolated DNA	Negative
*Clostridium butyricum* ATCC 19398	Isolated DNA	Negative
*Clostridium difficile* ATCC 9889	Boil prep DNA	Negative
*Clostridium sporogenes* ATCC 3584	Boil prep DNA	Negative
*Clostridium perfringens* ATCC 13124	Boil prep DNA	Negative
*Staphylococcus epidermidis* ATCC 12228	Isolated DNA	Negative
*Staphylococcus aureus* ATCC13565	Isolated DNA	Negative
*Listeria monocytogenes*	Isolated DNA	Negative
*Listeria innocua*	Isolated DNA	Negative
*Salmonella Typhimurium* ATCC 14028	Isolated DNA	Negative
*Escherichia coli* O157:H7 strain EDL933	Isolated DNA	Negative
Norovirus G11. 4	Isolated RNA	Negative
Hepatitis A strain HM175	Isolated RNA	Negative

DNA from other important food and waterborne protozoan parasites was also purchased or extracted (Table 2) in order to evaluate the specificity of the *Cyclospora* LAMP assay. *Eimeria acervulina* oocysts in infected chicken faeces were obtained from Dr. Rebecca Guy, Public Health Agency of Canada. DNA was extracted from the *Eimeria*-positive chicken faeces using a Qiagen DNeasy® Blood and Tissue Kit (Qiagen, Toronto, ON, Canada) as per manufacturer's instructions in addition to freeze-thaw cycling. The DNA concentration was 83.95 ng/μL as determined using a BioDrop spectrophotometer (MBI, Dorval, QC). *Giardia duodenalis* DNA (strain WB) (ATCC 30957D), was purchased at a concentration of 4.280 ng/μL. *Cryptosporidium parvum* DNA (ATCC PRA-67DQ) was purchased at a concentration of 3.590 ng/μL. *Toxoplasma gondii* DNA (strain RH) (ATCC 50174D) was purchased at a concentration of 5.260 ng/μL. *Giardia*, *Cryptosporidium* and *Toxoplasma* DNA was purchased from Cedarlane Labs (Burlington, ON).

## 3 Results

The novel LAMP assay described here for the detection of *C. cayetanensis* DNA in stool specimens was rapid, sensitive and specific. This LAMP assay enabled the detection of *C. cayetanensis* DNA within 30 min. It was also found to be specific to *C. cayetanensis* as it did not amplify DNA from a closely related coccidian parasite *Eimeria acervulina*, nor from several other important foodborne pathogens (Table 2).

**Fig. 1:**
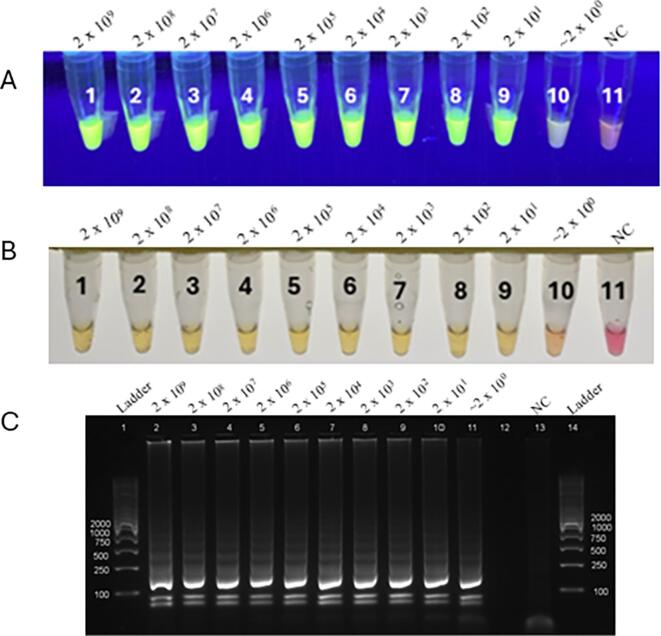
Detection of Cyc-Mit gBlock positive control DNA by fluorescent and colorimetric LAMP. (A) SYBR green I, where green indicates a positive reaction. (B) Phenol red, where yellow indicates a positive reaction. In both cases, lanes 1–10 = serial dilutions of the Cyc-Mit gBlock from 2 × 10^9^ copies to ∼2 × 10^0^ copies per reaction, lane 11 = negative control. (C) Reaction products visualized via agarose gel to confirm amplification. Lanes 1 and 14 = DNA Ladder (GelPilot Mid Range Ladders), lanes 2–11 = serial dilutions of the Cyc-Mit gBlock from 2 × 10^9^ copies to ∼2 × 10^0^ copies per reaction, lane 12 = not used, lane 13 = negative control. (For interpretation of the references to colour in this figure legend, the reader is referred to the web version of this article.)

Under UV light, the LAMP products of Cyc-Mit gBlock stained with SYBR Green I fluoresced at all dilutions down to 1:10^8^ (2 × 10^1^ copies) while no fluorescence was observed in the negative control (Fig. 1A). Similarly, using phenol red, the LAMP products could be directly visualized down to a 1:10^9^ dilution (∼2 × 10^0^ copies) (Fig. 1B). When the LAMP products of Cyc-Mit gBlock were electrophoresed and visualized on an agarose gel, bands were clearly visible down to a 1:10^9^ dilution (∼2 × 10^0^ copies) (Fig. 1C).

**Fig. 2:**
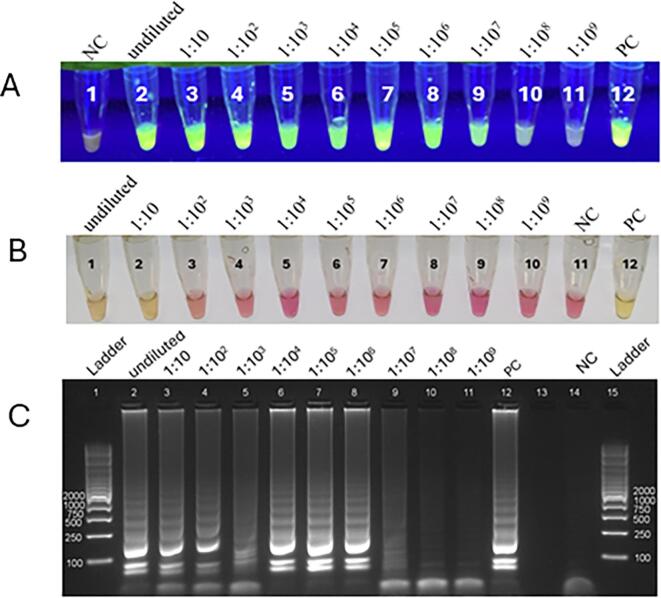
Detection of *Cyclospora cayetanensis* in stool by fluorescent and colorimetric LAMP. (A) SYBR green I, where green indicates a positive reaction, lane 1 = negative control, lanes 2–11 = serial 1:10 dilutions of the DNA extracted from the stool specimen, lane 12 = positive control. (B) Phenol red, where yellow indicates a positive reaction, lanes 1–10 = serial 1:10 dilutions of the DNA extracted from the stool specimen, lane 11 = negative control, lane 12 = positive control. (C) Reaction products visualized via agarose gel to confirm amplification. Lanes 1 and 15 = DNA Ladder (GelPilot Mid Range Ladders), lanes 2–11 = serial 1:10 dilutions of the DNA extracted from the stool specimen, lane 12 = positive control, lane 13 = not used, lane 14 = negative control. (For interpretation of the references to colour in this figure legend, the reader is referred to the web version of this article.)

Using DNA extracted from the positive stool specimen, LAMP products stained with SYBR Green I fluoresced down to at least a 1:10^7^ dilution, while no fluorescence was observed in the no-template negative control (Fig. 2A) nor from the *Cyclospora*-negative stool specimen (not shown). Conversely, using phenol red, LAMP products could only be visualized down to a 1:10 dilution (Fig. 2B). When the LAMP products were electrophoresed and visualized on an agarose gel, bands were clearly visible down to at least a 1:10^6^ dilution (Fig. 2C). Results shown in these figures are representative of three replicates which demonstrated very consistent levels of sensitivity.

## 4 Discussion

Loop-mediated isothermal amplification has been used successfully in detecting a number of human pathogens, including important food and waterborne protozoan parasites. The present study, however, is the first to report on the development of a LAMP assay with possible applications for the detection of *Cyclospora cayetanensis* DNA in food, environmental, and clinical samples.

The LAMP assay described here was found to be rapid, sensitive and specific in the detection of *C. cayetanensis* DNA in a stool specimen. This assay could be completed in only 30 min, making it considerably faster than PCR which can take up to 2 h and requires specialized equipment. LAMP has the added advantage of not being affected by DNA polymerase inhibitors, or various other biological substances which may be present in clinical and environmental samples, and which may inhibit PCR reactions (Kaneko et al., 2007; Zhang et al., 2019).

This LAMP assay was also found to be specific to *C. cayetanensis*, as it did not amplify DNA from other important food and waterborne pathogens, including *Giardia duodenalis*, *Cryptosporidium parvum*, and *Toxoplasma gondii*. Furthermore, this assay did not amplify DNA from the closely related coccidian parasite *Eimeria* spp. (Relman et al., 1996). Although *Eimeria* species are not human pathogens; their oocysts are often present in the environment. As a result; there is a potential for false *Cyclospora*-positive results in surveillance studies and outbreak investigations; as may occur when using PCR to amplify a region of the small subunit rRNA gene since the same size product is amplified from *Eimeria* species (Jinneman et al., 1998; Orlandi et al., 2003).

Recently, Barratt et al. (2023) described two novel species of *Cyclospora* as aetiological agents of human cyclosporiasis (i.e.; *C. ashfordi* and *C. henanensis*). Using MEGA X software we were able to identify and align consensus sequences among 35 different *C. cayetanensis* isolates from the NCBI database; some of which represent the two novel species. As there were only a small number of SNPs among the entire mitochondrial genomes; and 100% homology within the primer binding region; we can conclude that our LAMP assay will also target these novel species. Of the 22 named species of *Cyclospora* (Zhang et al., 2021), including both human and animal species, the mitochondrial genomes of only two non-human infectious species are available on NCBI and, while they generally show variation from the *C. cayetanensis* genomes, the amplified region is identical indicating that they would likely be amplified by our LAMP assay as well. Further study will be needed, therefore, to confirm whether our LAMP assay is genus or species specific.

This LAMP assay was found to be sensitive in the detection of *C. cayetanensis* DNA, particularly as determined using UV visualization with SYBR Green I dye, with DNA in the synthetic gene fragment being detected down to a dilution factor of 1:10^8^ (2 × 10^1^ copies) and DNA in a confirmed positive stool specimen being detected down to a dilution factor of at least 1:10^7^.

Colorimetric visualization of LAMP products from the stool specimen showed a lower sensitivity than UV visualization. Unlike SYBR Green I, which binds directly to the double-stranded DNA produced during the LAMP reaction, phenol red is simply a pH indicator which signals a drop in pH with the amplification of DNA. As a result, the sensitivity of phenol red in visualizing LAMP products may be reduced when working with complex matrices such as faeces where the pH is variable and which may contain various metabolic byproducts. For this reason, it is recommended that UV visualization of LAMP products, using a fluorescent dye such as SYBR Green I, be used when testing stool specimens.

There are a few important limitations with LAMP assays, including susceptibility to DNA contamination from the laboratory environment (as well as to the laboratory environment following amplification), potential for non-specific amplification, challenges in primer design, the inability to check for reaction inhibitors, and the inability to sequence the products (Kim et al., 2023; Soroka et al., 2021; Wong et al., 2018; Yang et al., 2024). Most of these limitations can be readily addressed however. DNA contamination of the laboratory environment, for example, can occur during the addition of SYBR Green I following LAMP amplification as this requires opening the tube (Wong et al., 2018). We were able to circumvent this issue by adding a droplet of concentrated SYBR Green I to the inside cap of the amplification tube prior to amplification. By mixing the SYBR Green I with the master mix following the incubation; we also avoided any dye inhibition of the LAMP amplification (Quyen et al., 2019). Non-specific amplification can be addressed through the use of loop primers; which greatly increase the specificity of the assay as well as the sensitivity and speed (Soroka et al., 2021), and LAMP primer design software is now readily available online. One other potential limitation with LAMP is that with the use of six primers, as compared to only two in PCR, mismatches may be more likely. For this reason, a highly conserved region of the *C. cayetanensis* mitochondrial genome was chosen when developing our assay. Alignments of all 35 available mitochondrial genomes showed no mismatches within any primer binding site. Even if minor mismatches were to occur within novel isolates, they would likely have only a negligible effect on the speed and sensitivity of the assay.

Although LAMP products cannot be directly sequenced, DNA in samples found to be positive by LAMP can be readily amplified using conventional PCR and the amplicons subsequently sequenced. As such, LAMP should be considered a rapid screening tool to be followed up by further molecular analyses for confirmation and genotyping.

In this study, we have developed a rapid, cost-effective, sensitive and specific LAMP assay for the detection of *C. cayetanensis* DNA. The findings of this study suggest that LAMP may be a more accessible and useful detection method than PCR in low-income countries and in point-of-care testing. Work is currently underway in our laboratory to validate this LAMP assay against a recently described qPCR assay targeting the *C. cayetanensis* mitochondrial gene Mit1C (Balan et al., 2023). Subsequent work will be done to determine the effectiveness of this LAMP assay in the detection of *C. cayetanensis* in spiked fresh produce and water samples. As LAMP assays are isothermal; and products can be immediately visualized; they have recently been recognized as being suitable for use with microfluidic technologies (Chen et al., 2023; Malic et al., 2022; Yang et al., 2024; Zhang et al., 2019). As such, we are also currently evaluating the effectiveness of LAMP assays for use in a microfluidic lab-on-a-chip device for point-of-care testing for *C. cayetanensis* and other important foodborne protozoan parasites.

## CRediT authorship contribution statement

**Mahdid Meymandy:** Visualization, Validation, Methodology, Formal analysis, Writing – review & editing. **Nadine Adam:** Visualization, Validation, Methodology, Writing – review & editing. **Nathalie Corneau:** Supervision, Resources, Methodology, Writing – review & editing. **Brent R. Dixon:** Supervision, Resources, Project administration, Investigation, Conceptualization, Writing – original draft.

## Declaration of competing interest

The authors declare the following financial interests/personal relationships which may be considered as potential competing interests:

Advisory board member of FAWPAR - Brent Dixon. Given his role as advisory board member, Brent Dixon had no involvement in the peer review of this article and had no access to information regarding its peer review. Full responsibility for the editorial process for this article was delegated to another journal editor. If there are other authors, they declare that they have no known competing financial interests or personal relationships that could have appeared to influence the work reported in this paper.
