# Supplementary material for: Development of a loop-mediated isothermal amplification (LAMP) assay for the detection of Cyclospora cayetanensis
Source: Food Waterborne Parasitol. 2026 May 5;43:e00337. doi: 10.1016/j.fawpar.2026.e00337 (PMC13185916; doi:10.1016/j.fawpar.2026.e00337)
Supplement: Supplementary file 1 — Supplementary material [file mmc1.docx]

Supplementary Table 1. Accession numbers for the 35 *Cyclospora cayetanensis* mitochondrial genomes available on NCBI

CM003498.1

CM034245.1

CM037477.1

CM075944.1

KP231180.1

KP658101.1

KP796149.1

MG831586.1

MG831587.1

MG831588.1

MN260345.1

MN260346.1

MN260347.1

MN260348.1

MN260349.1

MN260350.1

MN260351.1

MN260352.1

MN260353.1

MN260354.1

MN260355.1

MN260356.1

MN260357.1

MN260358.1

MN260359.1

MN260360.1

MN260361.1

MN260362.1

MN260363.1

MN260364.1

MN260365.1

MN260366.1

MN316534.1

MN316535.1

NC_038230.1

Supplementary Table 2. Sequence of the Cyc-Mit gBlocks® Gene Fragment, a 501 bp fragment from the *C. cayetanensis* mitochondrial genome region (3,300-3,800 bp)

| 5' - TTC AAA TAG ATG TAT TTT TTT GGC TAG AGT ACG TAA GGA AAA GGA AAG GTT AAC CGC TGT CAA AAA CAA AAA CTT ATT ACA TAC ACT CCT GAA CAT TTG GAA CAT ATG GAT ATA ATT TGG TAG TGG AAC ATT TGA AAT AAA AAT TGG CAG CTG GAA GAC GGA ATC GTT ACT AAG CGT CAG GTA GTC CTG GAC ACT GAA TCC ATG CGT GAT CCC AAT GGA ACG GTC CCT GGC TGA ATT TTA TGA TCC CAG GCT GGT TCA AAA AGT CAA ATA TTA GCC AAA AAC TGG CGA GAA GGG AAG TGT GTT TCT TAG ACA AAA AAT AAG GGA AGT TTA GCC GGG AAG TTA GCG TCT AAA AAA TAT GAT ATA ATT ATC GCA CAA GCA CTC AGC AAG TTA AGA GAA TGT ATT GAC GTG TTA AAA ACC GGT TTT TTG TTA GGG TGC CGG GCA GAT GTC ATA AAC TAT ACC TCC TCA TCA AAG TTA GCA GTG TCT TAC GTT TGA ATC CAA CAG - 3’ |
| --- |
